# Evaluation of Mchare and Matooke Bananas for Resistance to *Fusarium oxysporum* f. sp. *cubense* Race 1

**DOI:** 10.3390/plants9091082

**Published:** 2020-08-23

**Authors:** Privat Ndayihanzamaso, Diane Mostert, Megan Ceris Matthews, George Mahuku, Kennedy Jomanga, Happyness Justine Mpanda, Hassan Mduma, Allan Brown, Brigitte Uwimana, Rony Swennen, Robooni Tumuhimbise, Altus Viljoen

**Affiliations:** 1 Department of Plant Pathology, Stellenbosch University, Private Bag X1, Matieland 7602, South Africa; ndayihanzamasoprivat@gmail.com (P.N.); megandutoit@sun.ac.za (M.C.M.); altus@sun.ac.za (A.V.); 2 International Institute of Tropical Agriculture (IITA) Regional Hub, Plot 25, Light Industrial Area, Coca Cola Rd, P.O. Box 34441, Dar es Salaam, Tanzania; g.mahuku@cgiar.org; 3 International Institute of Tropical Agriculture (IITA), c/o The Nelson Mandela African Institution of Science and Technology (NM-AIST), P.O. Box 447, Arusha, Tanzania; kennedyjomanga@gmail.com (K.J.); happympanda91@gmail.com (H.J.M.); h.mduma@cgiar.org (H.M.); a.brown@cgiar.org (A.B.); rony.swennen@kuleuven.be (R.S.); 4 International Institute of Tropical Agriculture (IITA), Namulonge, P.O. Box 7878, Kampala, Uganda; b.uwimana@cgiar.org; 5 Laboratory of Tropical Crop Improvement, Katholieke, Universiteit Leuven (KUL), Willem De Croylaan 42, Bus 2455, 3001 Leuven, Belgium; 6 Bioversity International, Willem De Croylaan 42, 3001 Leuven, Belgium; 7 National Agricultural Research Organization (NARO), Rwebitaba ZARDI, P.O. Box 96, Fort Portal, Uganda; rtumuhimbise@hotmail.com

**Keywords:** East African Highland bananas, Fusarium wilt, resistance screening

## Abstract

Fusarium wilt, caused by the soil-borne fungus *Fusarium oxysporum* f. sp. *cubense* (Foc) race 1, is a major disease of bananas in East Africa. Triploid East African Highland (Matooke) bananas are resistant to Foc race 1, but the response of diploid (Mchare and Muraru) bananas to the fungus is largely unknown. A breeding project was initiated in 2014 to increase crop yield and improve disease and pest resistance of diploid and triploid East African Highland bananas. In this study, eight Mchare cultivars were evaluated for resistance to Foc race 1 in the field in Arusha, Tanzania. In addition, the same eight Mchare cultivars, as well as eight Muraru cultivars, 27 Mchare hybrids, 60 Matooke hybrids and 19 NARITA hybrids were also screened in pot trials. The diploid Mchare and Muraru cultivars were susceptible to Foc race 1, whereas the responses of Mchare, NARITAs and Matooke hybrids ranged from susceptible to resistant. The Mchare and Matooke hybrids resistant to Foc race 1 can potentially replace susceptible cultivars in production areas severely affected by the fungus. Some newly bred Matooke hybrids became susceptible following conventional breeding, suggesting that new hybrids need to be screened for resistance to all Foc variants.

## 1. Introduction

Banana cultivation in East and Central Africa (ECA) is dominated by a group of cooking bananas commonly referred to as East African Highland bananas (EAHB). These bananas were introduced into the region from Asia about 2500–3000 years ago, where after secondary evolution took place in the highlands of Uganda and Tanzania [1,2]. Today, EAHBs sustain the livelihoods of millions of small-scale farmers in ECA [3,4]. Their productivity is, however, low due to biotic stresses such as Fusarium wilt, Sigatoka leaf diseases, Xanthomonas wilt, nematodes weevils, and abiotic constraints such as declining soil fertility and drought. To overcome these stresses, banana in the region needs genetic improvement [5,6].

EAHBs comprise diploid and triploid bananas [1,7,8,9,10]. The diploids (AA) include Mchare bananas that are grown in the Kilimanjaro and Arusha (north-central) areas of Tanzania, and in the Mbeya (southern highlands) region, whereas the Muraru bananas are grown at the foothills of Mount Kenya, Mlali bananas in Mayotte and the Comoros Islands, and Paka bananas in Zanzibar [7,10,11,12]. Mlali bananas are also found in other regions of Tanzania such as Morogoro and Bukoba [11]. Mchare, Muraru and Mlali bananas are genetically similar, although phenotypic traits can distinguish them from each other [10]. While these diploids are mostly cooking types, Muraru bananas are consumed as dessert bananas in Kenya [11]. Triploid EAHBs (AAA), which are genetically uniform [9,13], include the Matooke (cooking) and Mbidde (juice/beer) bananas in the Lujugira-Mutika subgroup [14]. Matooke and Mbidde are widely grown throughout the ECA region [7,11,12].

Fusarium wilt of banana, a disease caused by the soil-borne fungus *Fusarium oxysporum* f. sp. *cubense* (Foc), is present in most banana-growing regions of ECA [15]. Foc infects banana plants through the roots and colonizes the vascular tissues to prevent water and nutrients from reaching the aerial parts [16,17]. This results in the yellowing and wilting of leaves and, ultimately, death of the entire banana plant [16]. Foc produces chlamydospores that can survive in the soil for many years, making it impossible to control [16,17,18]. The fungus spreads with infected planting material and soil attached to shoes, vehicles and plantation equipment [16,19,20,21,22,23]. The only means to deal with Fusarium wilt is to prevent the introduction of the fungus into banana fields, and to plant Fusarium wilt-resistant varieties [24].

Foc is diverse and comprises three races based on their pathogenicity to a group of differential cultivars, with Foc races 1, 2 and 4 causing disease to Gros Michel, Bluggoe and Cavendish bananas, respectively [25,26]. All three races are present in Africa, but only Foc races 1 and 2 have been reported in ECA [22,27,28]. Foc strains belonging to the two races are phylogenetically related and group together as Foc Lineage VI [29,30]. Fusarium wilt in ECA was first reported in Tanzania, Uganda and Kenya in the 1950s [16,19,20,21,26]. Cultivars susceptible to Foc race 1 include Sukari Ndizi (Apple banana, AAB) and Kayinja (Pisang Awak, ABB), with Foc race 2 affecting Bluggoe (ABB) bananas [22]. Matooke bananas are resistant to Foc races 1 and 2 [31,32], but diploid EAHBs have only been partially evaluated. Seven Mchare cultivars maintained at Kawanda in Uganda were found to be susceptible to Foc race 1 in a screen house evaluation [33], but there are no scientific reports of Fusarium wilt of Mchare bananas in the field in Uganda and Tanzania. The disease has, however, been reported on a close relative of Mchare bananas, the Muraru banana [34]. 

Bananas can be evaluated for Fusarium wilt resistance in pot assays under screen house and greenhouse conditions, or in the field. Results obtained from pot and field screenings are not always similar. Field screenings provide an evaluation of plant response under prevailing environmental and geographic conditions, but depend on the distribution of inoculum in the soil and the Foc strain present [35]. Pot assays are conducted under controlled environments, but are affected by Foc inoculum levels, the age of plantlets, temperature, the soil and the inoculation method used [20,36,37,38]. It is, therefore, important that the small plant techniques provide an accurate account of the banana plant’s field response. 

In 2014, a project to breed EAHB for higher yields and for disease and pest resistance started in Uganda and Tanzania. Mchare hybrids were derived from crosses between Mchare cultivars and diploid bananas resistant to Foc race 1 [39]. Nineteen NARITA and 60 Matooke hybrids were also developed from crosses between Matooke bananas and diploid bananas. The NARITA hybrids were jointly developed by the National Agricultural Research Organization (NARO) of Uganda and the International Institute of Tropical Agriculture (IITA) for high yield and black Sigatoka resistance, and were a result from crossing tetraploid females (triploid Matooke x Calcutta 4) and improved diploid males [40,41]. It was hypothesised that some of the Mchare hybrids would be more resistant to Foc race 1 than the parent cultivars, and that some NARITA and Matooke hybrids might be susceptible due to the breeding parents used. The first objective of the current study, was to evaluate eight Mchare (AA) cultivars in pot and field trials for resistance to Foc race 1 to determine the reliability of pot trials to estimate field resistance. Pot trials were then used to evaluate Muraru cultivars and Mchare, NARITA and Matooke hybrids.

## 2. Results

### 2.1. Evaluation of Mchare Cultivars in Arusha, Tanzania, against Foc Race 1

#### 2.1.1. Screen House Evaluation

All Mchare cultivars and Sukari Ndizi, the susceptible control, developed internal rhizome symptoms of Fusarium wilt in the screen house 10 weeks after inoculation with Foc race 1 with disease incidence between 88.9–100% (Table 1). The disease was slow to develop on the Mchare cultivars compared to Sukari Ndizi, but the cultivars were severely affected at the end of the trial with a rating of at least 3 on a scale of 1 to 6. Fusarium wilt symptoms that developed on Mchare Laini and Makyughu II did not differ significantly from Sukari Ndizi at the end of the pot trial. Grande Naine, the resistant control, did not develop Fusarium wilt symptoms, and was significantly more resistant than all the Mchare cultivars and Sukari Ndizi (Table 1).

#### 2.1.2. Field Evaluation

Makyughu II, Mchare Laini, Akondro Mainty, Mchare Mlelembo, Huti Green Bell and Ijihu Inkundu developed internal rhizome discolouration in the plant crop at Arusha, with a RDI that did not differ significantly from that of the susceptible control, Sukari Ndizi (Table 1). Huti White, Kahuti, and the resistant control variety Nakitengwa, did not show any internal rhizome symptoms in the plant crop, but displayed external leaf yellowing symptoms typical of Fusarium wilt, as indicated by the area under the disease progress curve (AUDPC) (Table 1, Figure S1). Leaf yellowing symptoms in the developed substantially slower than that of the susceptible control Sukari Ndizi (Figure S1). In the first ratoon, all the Mchare cultivars developed internal rhizome symptoms, and the RDI and AUDPC differed significantly from that of Nakitengwa, the resistant control (Table 1). They were, also, significantly less susceptible to Foc race 1 than Sukari Ndizi. The disease incidence for the susceptible control in both production cycles was 98.1%, while it ranged from 58.5–98.1% for the Mchare cultivars, except for Huti White and Kahuti that did not develop internal rhizome symptoms in the plant crop. 

#### 2.1.3. Correlation between Screen House and Field Evaluation

The RDI rating obtained for the screen house and field evaluation of Mchare cultivars was significantly positively correlated for the first ratoon (r = 0.76) (Table 2). This indicates that screen house testing could be used for the mass screening of banana accessions. 

### 2.2. Screen House Evaluation of Muraru Cultivars and Mchare Banana Hybrids against Foc Race 1 in Arusha, Tanzania

The Muraru cultivars were all susceptible to Foc race 1 with disease incidence between 88.9–100% and disease severity ranging from 3.7 for Muraru M3 to 5.8 for Njuru, compared to the 6.0 for the susceptible Sukari Ndizi (Table 3). Disease severity for all the cultivars differed significantly from the resistant Nakitengwa control. Rhizome discolouration of Muraru White, Mraru Mchare, TTZ 4 and Njuru cultivars were not significantly different from that of Sukari Ndizi, the susceptible control (Table 3). Although the disease severity in Muraru M3, Majimaji, Muraru Red and Mlalu was low compared to the susceptible control, the rating score was at least 3.7 on a scale of 6 (Table 3). Of the 23 Mchare hybrids, eight were considered resistant with disease incidence between 0–44.4%, five partially resistant with disease incidence between 66.7–100% and 10 susceptible to Foc race 1 with disease incidence between 88.9–100% (Table 3).

### 2.3. Screen House Evaluation NARITA Hybrids against Foc Race 1 in Kawanda, Uganda

Nineteen NARITA hybrids were evaluated in the screen house. Of these, only NARITA 16 and NARITA 12 developed Fusarium wilt symptoms (disease incidence between 55.6–88.9%), with disease severity significantly higher than those in Mbwazirume, the resistant control, but also significantly less than those in Sukari Ndizi, the susceptible control (Table 4). NARITA 16 and NARITA 12, therefore, could be considered as partially resistant. Some NARITA 7, NARITA 11 and NARITA 4 plants developed internal rhizome symptoms (disease incidence between 11.1–22.2%), but average disease severity was not different from that of the resistant control, and were thus ranked as resistant. None of the other NARITA hybrids developed Fusarium wilt symptoms, and were considered resistant (Table 4). 

### 2.4. Screen House Evaluation of Matooke Hybrids against Foc Race 1 in Kawanda, Uganda

Sixty Matooke hybrids were evaluated for resistance against Foc race 1. Thirty-eight of these were considered resistant, as their disease severity did not differ from that of Mbwazirume, the resistant control. Among the 38 resistant hybrids, 25 hybrids did not develop any Fusarium wilt symptoms (disease incidence 0%), whereas 13 hybrids had a few plants displaying internal spots in the inner rhizomes (disease incidence between 11.1–83.3%), with an average RDI below 2 on a scale of 6 (Table 5). Sixteen hybrids developed significantly more disease than Mbwazirume, the resistant control, but significantly less than Sukari Ndizi, the susceptible control, with disease incidence between 75–100%. They were grouped as hybrids with a partially resistance. The six remaining hybrids were considered susceptible because disease severity did not differ from that of Sukari Ndizi. Disease incidence in susceptible hybrids were between 88.9–100% (Table 5).

## 3. Discussion

Until recently, a limited amount of information was available on the response of diploid EAHB bananas to Foc race 1, despite the seasonal appearance of Fusarium wilt on Mchare and Muraru bananas in Tanzania and Kenya, respectively [33,34]. This lack of information delayed the management of banana Fusarium wilt in areas where losses occur, including efforts to improve EAHB diploids genetically for Fusarium wilt resistance. This study, therefore, provides the first comprehensive evaluation of diploid EAHB cultivars and hybrids for resistance to Foc race 1. It expands on the works of Arinaitwe et al. (2019) [33], which found seven Mchare cultivars were susceptible to Foc race 1 in screen house evaluations, and the report of Kung’U and Jeffries (2001) [34] which found a Muraru cultivar in Kenya to be affected by Fusarium wilt. Kung’U and Jeffries (2001) [34] also showed Muraru to be less susceptible to Foc race 1 (VCGs 0124 and 01220) than Bluggoe and Gros Michel bananas [34]. The resistant diploid EAHB cultivars identified in the current study, therefore, are potentially useful replacements for the susceptible cultivars in the area.

The results obtained from pot and field evaluations were well correlated, indicating that the pot assay can be used for the mass screening of banana varieties. Results obtained from pot trials when using the millet seed technique reflected those from field evaluation. The millet seed technique has also been widely used for the mass screening of banana varieties for Fusarium wilt resistance [33,43,44]. Foc-infected millet seeds were also successfully used for the field screening of banana for Fusarium wilt resistance [45].

Among the Mchare hybrids tested, hybrids obtained from the same parents (i.e., Huti White x Calcutta 4) did not produce the same results. T.2274-6 and T.2273-2, for instance, were resistant, while T.2269-2 and T.2691-15 were susceptible to Foc race 1. Calcutta 4 is resistant to Foc race 1 [46] and Huti White was found to be susceptible in this study, suggesting that the genes controlling resistance to Foc race 1 in the parent plants were heterozygous. These genes have most likely segregated in the gamete cells, resulting in different responses in the F_1_ offspring. An advanced screening of F_2_ population could confirm the nature of the inheritance of resistance. 

Matooke bananas are resistant to Foc race 1 [31,32]. NARITA 12 (Matooke hybrid), NARITA 16 (Matooke hybrid) and a number of newly developed Matooke hybrids were, however, susceptible to Foc race 1. The Matooke hybrids were not expected to be infected by Fusarium wilt because they resulted from crosses between resistant tetraploid Matooke hybrids and a diploid parent resistant to Foc race 1. For instance, NARITA 12 was partially resistant to Foc race 1, but its parents 1201 K-1 (Nakawere x Calcutta 4) and 9128-3 (Tjau Lagada x Pisang Lilin) are resistant [40]. Nakawere (Matooke), Calcutta 4, Tjau Lagada and Pisang Lilin are all bananas known to be resistant to Foc race 1 [31,47,48]. Although the Matooke cultivars are resistant to Foc race 1 and potentially to Foc tropical race 4 (TR4) [31,32], this study indicated that their hybrids can lose resistance following conventional breeding. This suggests that a number of genes confer resistance to Foc in Matooke bananas, and that these genes segregated during meiosis into gamete cells leading to loss in resistance of the newly bred Matooke hybrids. It is, for this reason, important for breeding programs to screen all new hybrids for resistance to all Foc variants.

The disease development in the Mchare cultivars Kahuti and Huti White at the field site in Arusha was slow compared to other Mchare cultivars. Makyughu II, Mchare Mlelembo, Akondro Mainty, Huti Green Bell and Ijihu Inkundu developed Fusarium wilt in the plant crop, but Kahuti and Huti White only showed internal rhizome symptoms during the first ratoon. It is unlikely that these varieties escaped the disease in the first production cycle, as diseased plants had been spread in the field during trial preparation. The delayed disease development might, however, be attributed to low inoculum levels or the uneven distribution of inoculum in the soil. If the cultivars were not evaluated for at least two production cycles, they would have been ranked as resistant. It is therefore important to consider increasing the inoculum in the field and/or to evaluate two production cycles when evaluating banana for disease resistance in the field. 

The evaluation of internal rhizome symptoms of Fusarium wilt near harvest proved to be of greater value than the rating of external leaf symptoms. Kahuti and Huti White varieties displayed yellowing of leaves that were rated as Fusarium wilt symptoms in the plant crop, but transverse cutting of rhizomes near harvest time showed no disease. This suggests that leaf yellowing may have been caused by other stresses. The rating of internal rhizome symptoms to assess Fusarium wilt is therefore needed when trial material presents little or no external symptoms. The leaf symptoms of Huti White and Kahuti that were visible in plant crop were probably caused by other stresses such as drought, deficiencies or nematodes, as the resistant control Nakitengwa also displayed a level of leaf yellowing.

In future, Mchare and Matooke hybrids with Foc race 1 resistance should be evaluated for agronomic performances and resistance to other important banana pests and diseases. Resistant diploids can also be used in banana breeding programs as sources of Foc race 1 resistance. Foc race 1-resistant EAHB hybrids should also be screened for resistance against Foc TR4. Foc TR4 has first been detected in northern Mozambique in 2013 (Viljoen, personal communication), and poses a serious threat to banana production in neighbouring Tanzania. It is known that banana cultivars respond differently to Foc race 1 and TR4, and that Foc TR4 has a larger host range [26,43,49,50].

## 4. Materials and Methods

### 4.1. Planting Materials

Eight Mchare cultivars and eight Muraru cultivars were evaluated for resistance to Foc race 1 in Arusha (Table 1 and Table 3), together with 23 Mchare hybrids (AA) from the IITA banana breeding programme in Tanzania. The Mchare cultivars are all endemic to the Arusha, Kilimanjaro and Mbeya regions in Tanzania. Nineteen NARITA hybrids (Table 4) and 60 Matooke hybrids from the IITA banana breeding programme in Uganda (AAA) (Table 5) were evaluated for resistance to Foc race 1 in Sendusu. The NARITA bananas are high-yielding Matooke hybrids that were developed in a joint breeding project by IITA and NARO. The plants were all tissue culture-derived, and were hardened-off for 2–3 months in screen houses before field and pot trial screenings. 

### 4.2. Fungal Isolates

Foc isolates CAV 3733 and CAV 3856, collected from infected bananas in Kawanda and Arusha, respectively, were used in Uganda and Tanzania as inoculum. The isolates were identified with Foc Lineage VI-specific DNA markers and by vegetative compatibility group (VCG) analysis. The Foc isolates were identified as Foc VCG complexes 0124/5/8/22 (CAV 3733) and 0124/22 (CAV 3856), respectively, which both belong to Foc race 1. The isolates are stored at the culture collection of Stellenbosch University’s Department of Plant Pathology. 

### 4.3. Preparation of the Inoculum

A millet seed technique was used to inoculate banana plantlets in screen house trials [51]. Finger millet (*Eleusine corocana*) seeds were washed and soaked in tap water for 6 h, the water was drained and the millet seeds autoclaved in 415 × 600 mm autoclavable bags (LabFriend Pty Ltd. Level, South Sydney, Australia) on two consecutive days for 20 min at 120 °C. Twenty to 30 mycelial plugs (10 mm × 10 mm) from a Foc race 1 isolate, grown on PDA for 4–5 days at 25 °C, were then used to inoculate 3 kg of sterile millet seeds in the autoclaved bags. The bags were incubated in the dark at 25 °C for 10–14 days and shaken every third day to ensure that the fungus properly colonised the millet seeds. 

### 4.4. Evaluation of Bananas for Resistance to Foc Race 1

#### 4.4.1. Pot Trials in Screen Houses

For screen house inoculations, 2-month-old banana plants were inoculated with Foc-colonised milled seed mixed into the potting soil. The eight Mchare cultivars, as well as the Matooke and NARITA hybrids, were planted in plastic pots containing 2 kg potting soil mixed with 20 g of the inoculum. The Muraru cultivars and Mchare hybrids, however, were replanted into 1 kg potting soil inoculated with 25 g of Foc-colonized millet seed. Before the greenhouse trials were conducted, a pilot study showed that the two inoculum concentrations gave the same result. After inoculation, the experiments were set up in a completely randomized block design (CRBD) with three plants per block, replicated three times. Thus a total of nine plants were used for each accession tested. 

In Arusha, Tanzania, Grand Naine (Cavendish, AAA) and Nakitengwa (EAHB) were used as resistant controls for the evaluation of Mchare cultivars, and Sukari Ndizi as the susceptible control (Table 1). For the Muraru cultivars and Mchare hybrids, however, only Nakitengwa and Sukari Ndizi were used as resistant and as susceptible controls, respectively (Table 3). In Sendusu, Uganda, Mbwazirume and Sukari Ndizi were used as resistant and susceptible controls, respectively, for the evaluation of NARITA and Matooke hybrids (Table 4 and Table 5). The 60 Matooke hybrids were evaluated in two groups, with the first group of 43 hybrids being inoculated in May 2019, and the second group of 17 hybrids in September 2019. 

For the screen house experiments plants were considered ready for rating when 50% of the susceptible control plants displayed leaf symptoms. This corresponded to a period ranging from 10–12 weeks after inoculation. During evaluation the rhizomes were cut open and the discolouration of inner rhizomes scored on a rating scale ranging from 1–6 [51].

#### 4.4.2. Field Evaluation

Field evaluation of Mchare banana cultivars for Foc race 1 resistance was conducted in Arusha, Tanzania. The land used was naturally infested by the fungus, and additional inoculum was added before the trial began by cutting up banana plants with Fusarium wilt symptoms, and spreading the diseased plant tissue across the trial site. Eight Mchare cultivars were then planted in a CRBD in April 2017, with 18 plants per cultivar in each block, replicated three times. (Table 1). Nakitengwa was included as the resistant control, and Sukari Ndizi as the susceptible control. No manure was applied to the field during or after planting, and weeding was done by hoeing. 

Disease in the field was monitored for two production cycles, known as the plant crop and the first ratoon. Disease progression in the field were scored using a 1–5 rating scale based on leaf discoloration [51]. The plant crop evaluation started 10 months after planting and disease was scored monthly for 7 months. The first ratoon evaluation started 24 months after the original planting, and disease was scored monthly for 6 months. At the end of the plant crop (17 months after planting) and first ratoon cycles (30 months after planting) respectively, the rhizomes of plants were cut open transversely, and internal symptoms scored on a rating scale of 1 to 6 [51]. When plants died before flowering or harvesting, they were scored as 6 (dead plants). A rhizome disease index (RDI) was the calculated as follows: Mid-percentages were assigned to each classification value. If RDI = 1 (no internal symptoms.); if RDI = 2 (mid-percentage = 5 indicating a few internal spots (<10%)); if RDI = 3 (mid-percentage = 25 with 10–33% of the rhizome discoloured); if RDI = 4 (mid-percentage = 50 with 33–66% of the rhizome discoloured); if a RDI = 5, (mid-percentage = 75 and 66–80% of the rhizome was discoloured); Finally, RDI = 6 (mid-percentage = 100 when the entire rhizome was discoloured and the plant was dead). A disease incidence was also calculated by dividing the number of plants displaying an internal rhizome discolouration of at least 2 by the total number of plants for each cultivar. Ten samples were collected from diseased plants and analysed to confirm infection by Foc race 1with Foc Lineage VI-specific PCR markers [52].

### 4.5. Data Collection and Statistical Analysis

An analysis of variance (ANOVA) was performed to compare Fusarium wilt resistance of the banana genotypes evaluated in the screen house and field trials, based on their RDI. XLSTAT software (XLSTAT, version 2018.1) was used to compute ANOVA, and multiple comparisons were performed between variables according to Fisher’s test at the 0.05 level of least significance difference (LSD). Pearson correlation test was performed between screen house and field screenings of Mchare cultivars in Arusha. The genotypes were ranked as ‘resistant’ when their RDI was less than ‘2’, as ‘partially resistant’ when it was between 2 and 3, and as ‘susceptible’ when the RDI was more than ‘3. To compare the progression of leaf yellowing in the field trial in Arusha, Tanzania, the AUDPC was measured [42] and an ANOVA performed.

The AUDPC was calculated as follows: AUDPC = $\sum_{i=1}^{n-1}\left(\frac{x_{i}+x_{i+1}}{2}\right)x\;\left(t_{i+1}-t_{i}\right)$, where *x_i_* is the severity of the disease observed at time *t_i_*, *x_i_*_+1_ the severity of the disease at the time of the subsequent evaluation *i* + 1, *t_i_* the time (months) at the time of observation *i*; *t_i_*_+1_ the time (months) at the time of the subsequent evaluation *i* + 1, and *n* the total number of evaluations. 

## Figures and Tables

**Table 1:** Screen house and field evaluation of Mchare cultivars (AA) for resistance to *Fusarium oxysporum* f. sp. *cubense* race 1 at Arusha, Tanzania.

Cultivar	ITC Code	Screen House Evaluation ^1^		Field Evaluation ^1^					
				Plant Crop			First Ratoon		
		Incidence (%) ^4^	Mean RDI ^2,^	AUDPC ^3^	Mean RDI ^2^	Incidence (%) ^4^	AUDPC ^3^	Mean RDI ^2^	Incidence (%) ^4^
Sukari Ndizi (Susceptible control)	-	100	6.0 a	18.9 a	4.5 a	98.1	14.7 a	3.9 a	98.1
Makyughu II	ITC 1446	100	5.1 ab	12.8 bc	3.3 a	73.6	10.4 bc	2.4 c	76.5
Mchare Laini	-	100	5.1 ab	14.3 b	2.5 ab	72.0	12.0 b	2.2 c	66.0
Huti White	-	100	4.9 b	10.2 cd	1.0 b	0	12.0 b	2.5 c	72.2
Kahuti	ITC 1468	100	4.8 b	8.6 de	1.0 b	0	9.9 c	2.3 c	58.5
Akondro Mainty	ITC 0281	100	4.3 b	8.8 de	2.7 ab	69.8	10.5 bc	3.1 b	77.4
Mchare Mlelembo	ITC 1455	88.9	4.2 b	10.1 cd	3.3 a	64.2	10.2 bc	2.5 bc	78.8
Huti Green Bell	ITC 1559	100	4.1 bc	10.2 cd	3.8 a	88.7	10.6 bc	2.4 c	73.1
Ijihu Inkundu	ITC 1460	88.9	3.0 c	11.9 bcd	3.8 a	98.1	10.5 bc	2.5 c	79.6
Grande Naine (Resistant control)		0	1.0 d	-	-	-	-	-	-
Nakitengwa (Resistant control)	ITC 0085		-	6.1e	1.0 b	0	5 d	1.0 d	0

^1^ Means with the same letter within the same column do not differ significantly according to Fisher’s test of least significant differences (*p* < 0.05). ^2^ The rhizome discolouration index (RDI) was scored on a rating scale ranging of 1 to 6, with 1 indicating no internal rhizome symptoms and 6 indicating the discolouration of the entire inner rhizome. RDI in the screen house was rated 10 weeks after inoculation, and in the field at the end of the plant crop and first ratoon production cycles. ^3^ The area under the disease progress curve was calculated using the formula developed by Shaner and Finney [42]. ^4^ Incidence was calculated as the mean percentage of plants affected by Fusarium wilt per cultivar.

**Table 2:** Correlation between screen house and field evaluation of Mchare cultivars for resistance to *Fusarium oxysporum* f. sp. *cubense* in Arusha, Tanzania.

	RDI Screen House	RDI Plant Crop	RDI First Ratoon
RDI Screen house	1		
RDI Plant crop	0.34 (*p* = 0.334)	1	
RDI First ratoon	**0.76** * (*p* = 0.010)	0.63 (*p* = 0.051)	1

* Values in bold indicate a significant correlation.

**Table 3:** Screen house evaluation of Muraru cultivars (AA) and Mchare hybrids (AA) for resistance to *Fusarium oxysporum* f. sp. *cubense* race 1 in Arusha, Tanzania.

Genotype	Parents	Incidence (%)	Mean RDI ^1,2,3^	Grouping
Controls				
Nakitengwa	Resistant control	0.0	1.0 l	Resistant
Sukari Ndizi	Susceptible control	100.0	6.0 a	Susceptible
Muraru cultivars		100.0		
Muraru M3	Landrace	100.0	3.7 gh	Susceptible
Majimaji	Landrace	100.0	4.4 defg	Susceptible
Muraru Red	Landrace	100.0	4.7 cdefg	Susceptible
Mlalu	Landrace	88.9	4.8 bcdef	Susceptible
Muraru White	Landrace	88.9	5.0 abcde	Susceptible
Mraru Mchare	Landrace	100.0	5.0 abcde	Susceptible
TTZ 4	Landrace	100.0	5.7 ab	Susceptible
Njuru	Landrace	100.0	5.8 ab	Susceptible
Mchare hybrids				
T.2327-1	Huti White x Cv Rose	0.0	1.0 l	Resistant
T.2274-6	Huti White x Calcutta 4	11.1	1.1 kl	Resistant
T.2273-2	Huti White x Calcutta 4	12.5	1.1 kl	Resistant
NM 185-1	Mchare Laini x Borneo	16.7	1.2 kl	Resistant
T.2274-12	Huti White x Calcutta 4	22.2	1.2 kl	Resistant
T.1768-1	Huti White x Calcutta 4	25.0	1.3 kl	Resistant
T.2274-3	Huti White x Calcutta 4	44.4	1.7 jkl	Resistant
T.2274-7	Huti White x Calcutta 4	44.4	1.9 jkl	Resistant
NM 211-1	Kahuti x Calcutta 4	100	2.0 jkl	Partially resistant
T.2003-1	Nshonowa x Calcutta 4	66.7	2.0 jkl	Partially resistant
T.2269-1	Huti White x Calcutta 4	77.8	2.0 jkl	Partially resistant
T.2070-1	Huti White x Borneo	77.8	2.1 jk	Partially resistant
T.2203-1	Nshonowa x Calcutta 4	100.0	2.4 ij	Partially resistant
NM 154-1	Kahuti x Borneo	100.0	3.3 hi	Susceptible
T.2691-9	Huti White x Calcutta 4	100.0	3.8 fgh	Susceptible
NM 226-11	Mchare Laini x Calcutta 4	100.0	4.1 efgh	Susceptible
T.2274-8	Huti White x Calcutta 4	100.0	4.3 defgh	Susceptible
T.2731-2	Mchare Laini x Borneo	88.9	4.4 defg	Susceptible
T.2269-2	Huti White x Calcutta 4	100.0	5.3 abcd	Susceptible
T.2691-15	Huti White x Calcutta 4	100.0	5.6 abc	Susceptible
NM 226-5	Mchare Laini x Calcutta 4	100.0	5.8 a	Susceptible
T.2317-1	Huti White x Borneo	100.0	6.0 a	Susceptible
NM 226-16	Mchare Laini x Calcutta 4	100.0	6.0 a	Susceptible

^1^ The rhizome discolouration index (RDI) was scored on a rating scale ranging of 1 to 6, with 1 indicating no internal symptoms and 6 indicating the discolouration of the entire inner rhizome. ^2^ Means with the same letter within the same column do not differ significantly according to Fisher’s test of least significant differences (*p* < 0.05). ^3^ RDI in the screen house was rated 10 weeks after inoculation.

**Table 4:** Screen house evaluation of NARITA hybrids (high-yielding selected Matooke hybrids) for resistance to *Fusarium oxysporum* f. sp. *cubense* race 1 in Kawanda, Uganda.

Genotype	Parents (Female x Male)	Pedigrees for the Female Parent	Pedigrees for the Male Parent	Incidence (%)	RDI ^1,2,3^	Grouping
Mbwazirume ^4^	Resistant control	-	-	0.0	1.0 c	Resistant
NARITA 3	917K-2 x SH3362	Enzirabahima x Calcutta 4	SH3217 x SH3142	0.0	1.0 c	Resistant
NARITA 8	917K-2 x SH3217	Enzirabahima x Calcutta 4	SH2095 x SH2766	0.0	1.0 c	Resistant
NARITA 9	917K-2 x SH3217	Enzirabahima x Calcutta 4	SH2095 x SH2766	0.0	1.0 c	Resistant
NARITA 10	917K-2 x SH3217	Enzirabahima x Calcutta 4	SH2095 x SH2766	0.0	1.0 c	Resistant
NARITA 13	1201K-1 x SH3362	Nakawere x Calcutta 4	SH3217 x SH3142	0.0	1.0 c	Resistant
NARITA 14	917K-2 x 7197-2	Enzirabahima x Calcutta 4	SH3362 x Long Tavoy	0.0	1.0 c	Resistant
NARITA 15	660K-1 x 9128-3	Enzirabahima x Calcutta 4	Tjau Lagada x Pisang Lilin	0.0	1.0 c	Resistant
NARITA 17	1438K-1 x 9719-7	Entukura x Calcutta 4	Madang x Calcutta 4	0.0	1.0 c	Resistant
NARITA 19	1201K-1 x 8075-7	Nakawere x Calcutta 4	SH3362 x Calcutta 4	0.0	1.0 c	Resistant
NARITA 20	Entukura x 365K-1	Unkown	Kabucuragye x Calcutta 4	0.0	1.0 c	Resistant
NARITA 21	1201K-1 x 7197-2	Nakawere x Calcutta 4	SH3362 x Long Tavoy	0.0	1.0 c	Resistant
NARITA 22	917K-2 x 9128-3	Enzirabahima x Calcutta 4	Tjau Lagada x Pisang Lilin	0.0	1.0 c	Resistant
NARITA 25	Unknown x Unknown	Unkown	Unknown	0.0	1.0 c	Resistant
NARITA 26	Unknown x Unknown	Unkown	Unknown	0.0	1.0 c	Resistant
NARITA 7	1201K-1 x SH3217	Nakawere x Calcutta 4	SH2095 x SH2766	11.1	1.1 c	Resistant
NARITA 11	1201K-1 x 9128-3	Nakawere x Calcutta 4	Tjau Lagada x Pisang Lilin	22.2	1.2 c	Resistant
NARITA 4	660K-1 x 9128-3	Enzirabahima x Calcutta 4	Tjau Lagada x Pisang Lilin	22.2	1.3 c	Resistant
NARITA 16	917K-2 x SH3362	Enzirabahima x Calcutta 4	SH3217 x SH3142	55.6	2.3 b	Partially resistant
NARITA 12	1201K-1 x 9128-3	Nakawere x Calcutta 4	Tjau Lagada x Pisang Lilin	88.9	2.7 b	Partially resistant
Sukari Ndizi ^4^	Susceptible control	-	-	100.0	4.1 a	Susceptible

^1^ The rhizome discolouration index (RDI) was scored on a rating scale ranging of 1 to 6, with 1 indicating no internal symptoms and 6 indicating the discolouration of the entire inner rhizome. ^2^ Means with the same letter within the same column do not differ significantly according to Fisher’s test of least significant differences (*p* < 0.05). ^3^ RDI in the screen house was rated 10 weeks after inoculation. ^4^ Mbwazirume and Sukari Ndizi were used as resistant and susceptible control respectively.

**Table 5:** Screen house evaluation of Matooke hybrids for resistance to *Fusarium oxysporum* f. sp. *cubense* race 1 in Kawanda, Uganda.

Genotype	Parents (Female x Male)	Genome Composition	Incidence (%)	Mean RDI ^1,2,3^	Grouping
Matooke hybrids evaluated in May 2019					
Mbwazirume ^4^	Resistant control	AAA	0.0	1.0 j	Resistant
29586S-4	1438K-1 x 5610S-1	AAA	0.0	1.0 j	Resistant
24948S-9	1438K-1 x 5610S-1	AAA	0.0	1.0 j	Resistant
29114S-24	5610S-1 x Malaccensis_250	AAA	0.0	1.0 j	Resistant
28256S-1	917K-2 x Cv. Rose	AAA	0.0	1.0 j	Resistant
28068S-9	917K-2 x Cv. Rose	AAA	0.0	1.0 j	Resistant
27579S-3	1201K-1 x Malaccensis_250	AAA	0.0	1.0 j	Resistant
27262S-1	1201K-1 x 9128-3	AAA	0.0	1.0 j	Resistant
26975S-2	917K-2 x 9128-3	AAA	0.0	1.0 j	Resistant
26666S-1	917K-2 x SH3362	AAA	0.0	1.0 j	Resistant
26316S-7	1201K-1 x SH3362	AAA	0.0	1.0 j	Resistant
26315S-1	1201K-1 x SH3142	AAA	0.0	1.0 j	Resistant
25974S-31	917K-2 x SH3362	AAA	0.0	1.0 j	Resistant
25499S-7	1438K-1 x SH3142	AAA	0.0	1.0 j	Resistant
25117S-1	917K-2 x 5610S-1	AAA	0.0	1.0 j	Resistant
26337S-11	1201K-1 x SH3217	AAA	0.0	1.0 j	Resistant
28476S-8	917K-2 x SH3362	AAA	11.1	1.1 ij	Resistant
28033S-23	917K-2 x Malaccensis_250	AAA	11.1	1.1 ij	Resistant
27873S-26	660K-1 x Malaccensis_250	AAA	11.1	1.1 ij	Resistant
28030S-2	1201K-1 xMalaccensis_250	AAA	22.2	1.2 ij	Resistant
25623S-11	917K-2 x 8817S-1	AAA	22.2	1.2 ij	Resistant
28492S-1	917K-2 x 1968-2	AAA	22.2	1.2 ij	Resistant
28260S-2	Enzirabahima x Calcutta 4	AAA	22.2	1.2 ij	Resistant
26990S-4	917K-2 x 5610S-1	AAA	22.2	1.2 ij	Resistant
28165S-1	1201K-1 x 1968-2	AAA	33.3	1.3 hij	Resistant
26337S-43	1201K-1 x SH3217	AAA	33.3	1.3 hij	Resistant
26337S-22	1201K-1 x SH3217	AAA	33.3	1.4 hij	Resistant
26337S-2	1201K-1 x SH3217	AAA	55.6	1.6 ghij	Resistant
25031S-17	5610S-1 x 2180K-6	AA	83.3	1.8 fghi	Resistant
27914S-1	1438K-1 x Cv. Rose	AAA	100.0	2.0 efgh	Partially resistant
25031S-7	5610S-1 x 2180K-6	AA	80.0	2.2 defg	Partially resistant
28260S-2	Enzirabahima x Calcutta 4	AAA	88.9	2.3 def	Partially resistant
28200S-3	917K-2 x Malaccensis_250	AAA	88.9	2.3 def	Partially resistant
25974S-30	917K-2 x SH3362	AAA	88.9	2.3 def	Partially resistant
27524S-22	917K-2 x Malaccensis_250	AAA	85.7	2.6 cdef	Partially resistant
27935S-1	1201K-1 x Cv. Rose	AAA	85.7	2.6 cdef	Partially resistant
28257S-1	917K-2 x Malaccensis_250	AAA	75.0	2.6 cde	Partially resistant
25583S-2	1201K-1 x 5610S-1	AAA	88.9	2.9 bcd	Partially resistant
27914S-18	1438K-1 x Cv. Rose	AAA	88.9	2.9 bcd	Partially resistant
27914S-7	1438K-1 x Cv. Rose	AAA	88.9	3.3 abc	Susceptible
28434S-9	1201K-1 x 5610S-1	AAA	88.9	3.4 ab	Susceptible
28776S-2	Tereza x 8075-7	AAA	88.9	3.6 ab	Susceptible
28246S-7	1201K-1 x Cv. Rose	AAA	100.0	3.7 a	Susceptible
Sukari Ndizi ^4^	Susceptible control	AAB	100.0	3.9 a	Susceptible
26260S-3	660K-1 x 5610S-1	AAA	100.0	4.0 a	Susceptible
Matooke hybrids evaluated in September 2019					
Mbwazirume ^4^		AAA	0.0	1.0 c	Resistant
25737S-1	917K-2 x 9128-3	AAA	0.0	1.0 c	Resistant
25435S-4	917K-2 x 9128-3	AAA	0.0	1.0 c	Resistant
29285S-20	1201K-1 x Cv. Rose	AAA	0.0	1.0 c	Resistant
28452S-11	Nakasabira x Calcutta 4	AAA	0.0	1.0 c	Resistant
27914S-26	1438K-1 x Cv. Rose	AAA	0.0	1.0 c	Resistant
25583S-2	1201K-1 x 5610S-1	AAA	0.0	1.0 c	Resistant
26337S-11B	1201K-1 x SH3217	AAA	0.0	1.0 c	Resistant
26337S-22B	1201K-1 x SH3217	AAA	0.0	1.0 c	Resistant
25974S-17	917K-2 x SH3362	AAAA	0.0	1.0 c	Resistant
29285S-20	1201K-1 x Cv. Rose	AAA	0.0	1.0 c	Resistant
27579S-1	1201K-1 x Malaccensis_250	AAA	55.6	2.0 b	Partially resistant
24948S-13	1438K-1 x 5610S-1	AAA	77.8	2.1 b	Partially resistant
27346S-4	1201K-1 x Malaccensis_250	AAA	77.8	2.3 b	Partially resistant
26840S-10	1201K-1 x SH3362	AAA	100.0	2.4 b	Partially resistant
24948S-10	1438K-1 x 5610S-1	AAA	87.5	2.4 b	Partially resistant
27770S-4	1201K-1 x Cv. Rose	AAA	100.0	2.6 b	Partially resistant
25328S-3	1438K-1 x 1537K-1	AAA	100.0	3.7 a	Susceptible
Sukari Ndizi ^4^	Susceptible control	AAB	100.0	4.2 a	Susceptible

^1^ The rhizome discolouration index (RDI) was scored on a rating scale ranging of 1 to 6, with 1 indicating no internal symptoms and 6 indicating the discolouration of the entire inner rhizome. ^2^ Means with the same letter within the same column do not differ significantly according to Fisher’s test of least significant differences (*p* < 0.05).^3^ RDI in the screen house was rated 10 weeks after inoculation. ^4^ Mbwazirume and Sukari Ndizi were used as resistant and susceptible control respectively.

## Supplementary Materials

The following are available online at https://www.mdpi.com/2223-7747/9/9/1082/s1, Figure S1: Field evaluation of Mchare cultivars for resistance to *Fusarium oxysporum* f. sp. *cubense* race 1 at Arusha, Tanzania.
