# Supplementary material for: Evaluation of Mchare and Matooke Bananas for Resistance to Fusarium oxysporum f. sp. cubense Race 1
Source: Plants (Basel). 2020 Aug 23;9(9):1082. doi: 10.3390/plants9091082 (PMC7570241; doi:10.3390/plants9091082)
Supplement: Supplementary file 1 [file plants-09-01082-s001.pdf]

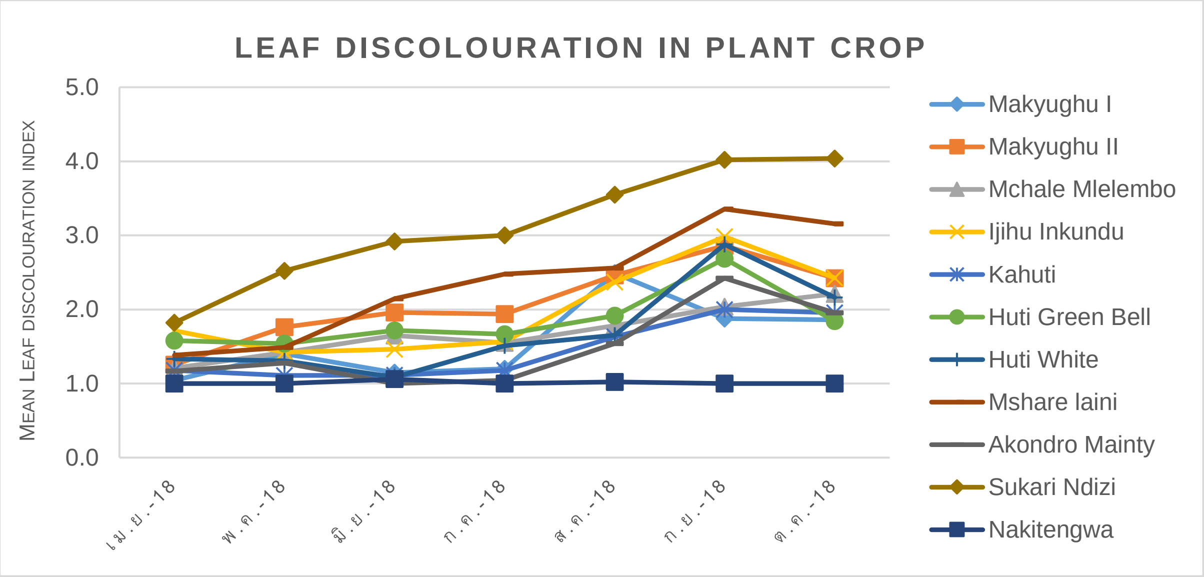

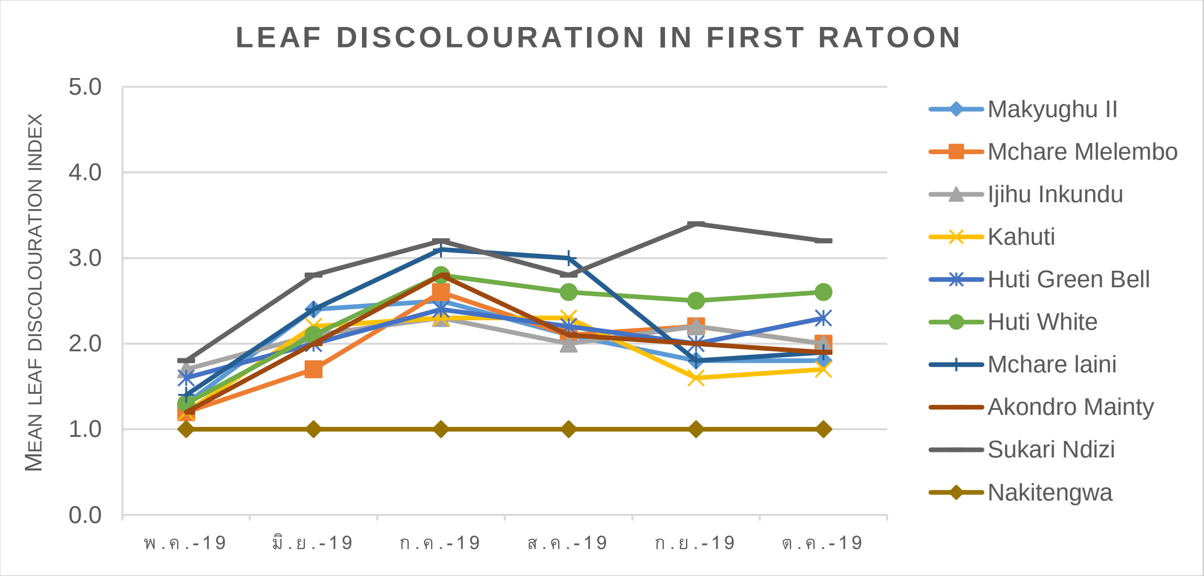

Figure S1. Field evaluation of Mchare cultivars for resistance to *Fusarium oxysporum* f. sp. *cubense* race 1 at Arusha, Tanzania. Each time point represents the average leaf discolouration on a rating scale of 1 to 5, with 1 indicating no leaf discolouration, and 5 indicating a dead plant.
